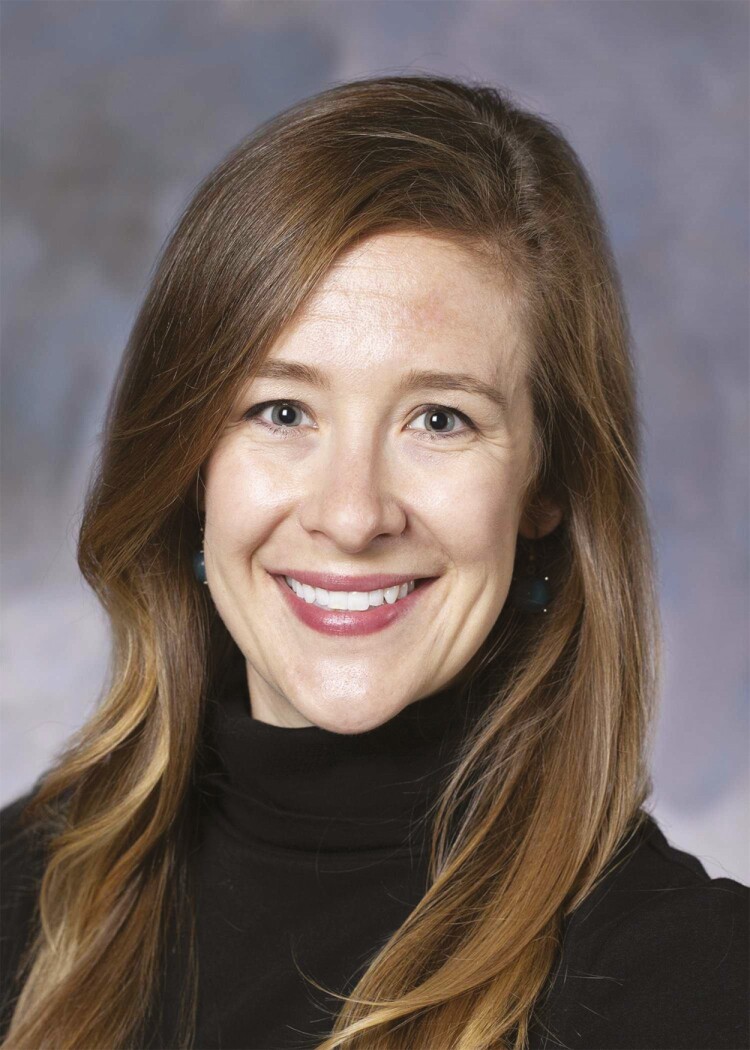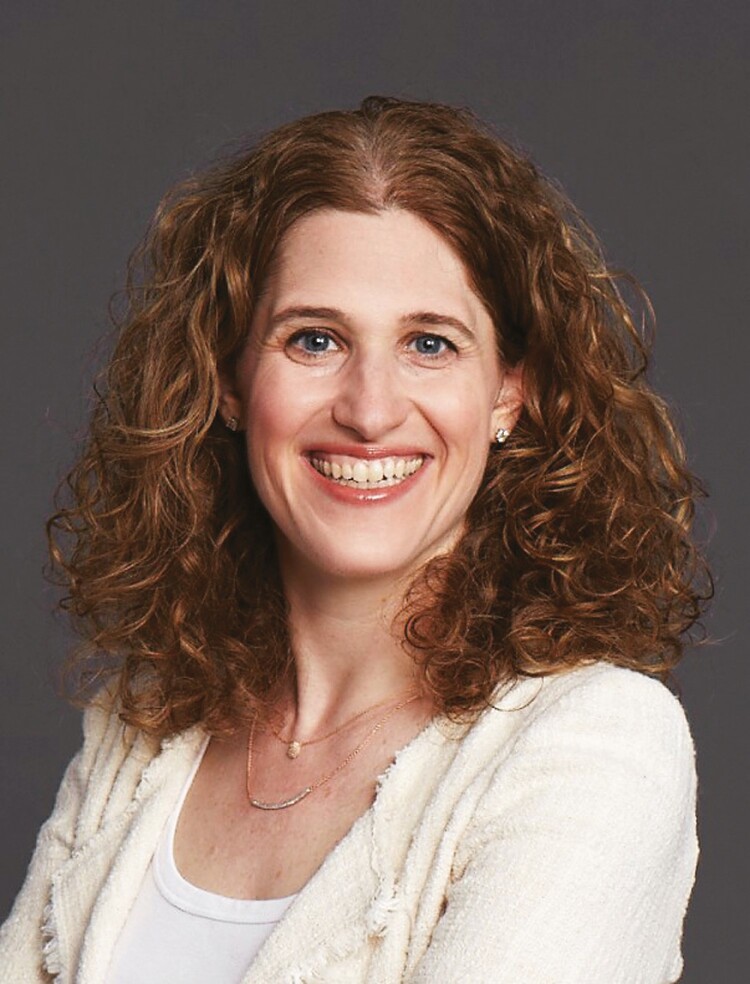# Training Pearl: Welcome to the CC360 Fellow’s Corner

**DOI:** 10.1093/crocol/otad060

**Published:** 2023-10-27

**Authors:** Hilary K Michel, Lisa Malter

**Affiliations:** Division of Gastroenterology, Nationwide Children’s Hospital, Columbus, OH, USA; Department of Pediatrics, The Ohio State University College of Medicine, Columbus, OH, USA; Department of Medicine, Division of Gastroenterology, NYU Langone Health, New York, NY, USA

**Keywords:** inflammatory bowel disease, medical education, fellowship, editor, reviewer

## Abstract

The Crohn’s & Colitis Foundation has grown to appreciate the needs of gastroenterology trainees with numerous initiatives designed to provide education, academic opportunities, and mentoring in inflammatory bowel disease (IBD) in recent years. The editors and staff at *Crohn’s and Colitis 360* (CC360) have launched 2 new initiatives, the Fellow’s Corner and the CC360 Editorial Fellowship, to support trainees in gaining knowledge and skills regarding peer review and publication as well as offering guidance on training in IBD and an opportunity for publication in this peer-reviewed, open access, quarterly online journal. These opportunities are described in this manuscript.

## Manuscript

No matter where one trains or in what setting one practices, engaging with research is part of a career in medicine. For some gastroenterologists (GIs), this may be the majority of their work: Devising research questions, designing projects to answer those questions, and analyzing and disseminating results. For clinically focused GIs, conducting research may not be the primary focus, but the ability to identify relevant research studies, interpret data, determine its validity, and apply it to clinical care is essential. Fellowship training is an ideal time to begin to develop skills in planning, conducting, and assessing research quality. Both pediatric and adult GI trainees completing a fellowship program accredited by the Accreditation Council for Graduate Medical Education are required to conduct scholarly activities during training which may include classic biomedical research, quality improvement, population health, and/or medical education, prior to their graduation.^1,2^ However, research-focused education and access to experienced research mentors vary among training programs as does education in inflammatory bowel disease (IBD).^3–5^ To help address this variability and support engagement of trainees in the research and publication process in IBD, *Crohn’s & Colitis 360* journal (CC360) created 2 new fellow-facing initiatives in 2023.

## The Fellow’s Corner

We have created and launched a new section within the Journal called The Fellow’s Corner, which you are now reading! Each issue of the journal will contain 2 articles, 1 Training Pearl and 1 original article first authored by a fellow. The Training Pearl will be authored by an experienced IBD clinician, and the majority of Training Pearls will be invited by the editorial board. Topics may include clinical skill development, medical education, and mentoring tips focused on scholarly productivity and career development in GI with a specific focus on those interested in IBD. The second publication in the Fellow’s Corner will be an opportunity to highlight original articles written by fellows. While a wide range of article types will be accepted for peer review, including commentaries or editorials on the topics of training, health policy, and advocacy, there is a preference for original research articles which, in some cases, may be considered for a waiver of publication fee. When submitting an original work to CC360, a box will be available to check in the submission portal that indicates whether the article should be considered for publication in the Fellow’s Corner. Manuscripts published in the Fellow’s Corner will also be cataloged and highlighted on the CC360 Fellow Initiatives webpage.

## *Crohn’s & Colitis 360* Journal Editorial Fellowship

The Editorial Fellowship is a 1-year, mentored experience designed for second- and third-year GI or IBD fellows. In this program, the Editorial Fellow collaborates with Associate Editors of CC360 to learn about the processes of peer review, editing, and publishing. Through reviewing manuscripts submitted to the journal and receiving feedback on these reviews from our Associate Editors, fellows hone their ability to identify strong research and provide constructive comments to authors. The program also provides the opportunity for networking within the IBD space, and for publication of peer-reviewed work in CC360. Applications will open for the 2024–2025 Editorial Fellow in the fall of 2023 and 1 fellow is selected per year to serve a term from February through the following January.

To be a well-rounded, effective GI, one must be both clinically savvy and knowledgeable about how to conduct, interpret, and review scholarly research. With these novel programs at CC360, we aim to support the development of trainees and contribute to the growing pipeline of academically excellent pediatric and adult GIs and IBD specialists. More information about these initiatives can be found here: https://academic.oup.com/crohnscolitis360/pages/360-fellows. If you have any questions, please do not hesitate to contact us at hilary.michel@nationwidechildrens.org and lisa.malter@nyulangone.org.